# HSFAN: A Dual-Branch Hybrid-Scale Feature Aggregation Network for Remote Sensing Image Super-Resolution

**DOI:** 10.3390/e27121189

**Published:** 2025-11-24

**Authors:** Jiawei Yang, Hongliang Ren, Mengjie Zeng, Zhichao He

**Affiliations:** College of Information Science and Engineering, Fujian Key Laboratory of Light Propagation and Transformation, Huaqiao University, Xiamen 361021, China; 24014082023@stu.hqu.edu.cn (J.Y.); 23014082031@stu.hqu.edu.cn (M.Z.); 24013082005@stu.hqu.edu.cn (Z.H.)

**Keywords:** super-resolution, multi-scale, remotely sensed imagery, information entropy

## Abstract

To address the issues of insufficient feature utilization in high-entropy regions (such as complex textures and edges), difficulty in detail recovery, and excessive model parameters with high computational complexity in existing remote sensing image super-resolution networks, a novel dual-branch hybrid-scale feature aggregation network (HSFAN) is proposed. The design of this network aims to achieve an optimal balance between model complexity and reconstruction quality. The main branch of the HSFAN effectively expands the receptive field through a multi-scale parallel large convolution kernel (MSPLCK) module, enhancing the ability to model global structures that contain rich information, while maintaining consistency constraints in the feature space. Meanwhile, an enhanced parallel attention (EPA) module is incorporated, optimizing feature allocation by prioritizing high-entropy feature channels and spatial locations, thereby improving the expression of key details. The auxiliary branch is designed with a multi-scale large-kernel attention (MSLA) module, employing depthwise separable convolutions to significantly reduce the computational overhead in the feature processing path, while adaptive attention weighting strengthens the capture and reconstruction of local high-frequency information. Experimental results show that, for the ×4 super-resolution task on the UC Merced dataset, the proposed algorithm achieves a PSNR of 27.91 dB and an SSIM of 0.7616, outperforming most current mainstream super-resolution algorithms, while maintaining a low computational cost and model parameter count. This provides a new research approach and technical route for remote sensing image super-resolution reconstruction.

## 1. Introduction

Remote sensing images are characterized by high information entropy, good durability, and wide coverage, making high-resolution remote sensing images valuable in environmental monitoring [1], land cover segmentation [2], hyperspectral image classification [3], change detection [4], and other research fields [5,6]. However, due to limitations in sensor hardware design and acquisition conditions, information loss often occurs during the acquisition and transmission of remote sensing images, resulting in low-resolution images. Enhancing image resolution by improving imaging hardware is difficult and costly. Therefore, exploring and reconstructing the potential information in images from an algorithmic perspective has become a mainstream approach to improving image resolution [7].

The principle of super-resolution (SR) is based on enhancing image spatial resolution. Essentially, it is a typical ill-posed inverse problem, which refers to recovering high-frequency information and details lost during the degradation process in high-resolution (HR) images from low-resolution (LR) images through certain mathematical models and algorithms [8]. Traditional super-resolution methods mainly rely on interpolation and reconstruction techniques, but these methods often fail to effectively handle the information bottleneck during the image degradation process and cannot fully exploit the high-level semantic information in images, resulting in reconstructed images with insufficient fidelity, lacking details and realism [9,10]. In recent years, with the rapid development of deep learning, deep learning-based image super-resolution methods have broken the limitations of traditional methods. Convolutional Neural Networks (CNNs) [11], with their powerful end-to-end learning capability and ability to capture complex image information mapping relationships, can learn efficient feature representations and information reconstruction from low-resolution images to high-resolution images, providing a completely new solution for image super-resolution problems.

In 2014, Dong et al. [12] first applied CNNs to the super-resolution problem, proposing the Super-Resolution Convolutional Neural Network (SRCNN), which achieved high-resolution image reconstruction using only three layers of CNNs, outperforming traditional methods in evaluation metrics such as PSNR and SSIM. Subsequently, various CNN-based algorithms have been proposed. For example, Kim et al. [13] introduced the Very Deep Super-Resolution Convolutional Network (VDSR), which increased the number of layers in the CNN to 20 and incorporated residual learning to address the issues of vanishing and exploding gradients. Lim et al. [14] proposed the Enhanced Deep Super-Resolution Network (EDSR), which extended the number of convolutional layers to 69 in order to extract deeper features. Mei et al. [15] designed a Non-Local Sparse Attention Network (NLSA) that improves performance using sparse non-local attention. These methods generally leverage CNN’s local connectivity and translational invariance, showing high efficiency and good generalization capability. However, their inherent architectural characteristics also lead to two major limitations: the limited receptive field makes it difficult to model long-range pixel dependencies, and the static convolution filters cannot fully adapt to the dynamic variations in input content. To overcome these limitations, some studies have combined CNN with Transformer architectures to enhance modeling capabilities. For example, the Swin Transformer for Image Restoration (SwinIR) [16] and the Efficient Long-range Attention Network (ELAN) [17] introduced self-attention mechanisms, effectively establishing global dependencies and long-range pixel correlations, compensating for the modeling deficiencies in CNN due to their local receptive fields and weight-sharing convolution kernels. Although these methods have made progress in capturing long-range dependencies, they overlook the interaction between channel and spatial dimensions, and fail to fully consider multi-scale features and the scale relationships among similar objects.

Despite the significant progress made by existing super-resolution methods, they still face the following three limitations: (1) Insufficient interaction between channels and spatial dimensions, as most of the methods ignore the effective interaction between channels and spatial dimensions. This makes them fail to fully utilize the potential dependencies between different dimensions when capturing image details and global semantic information. (2) Existing methods generally lack effective multi-scale information fusion and fail to fully consider the scale relationships between similar objects. This is especially important when dealing with remote sensing images, because remote sensing images often contain information at multiple scales, and single-scale feature extraction is unable to fully capture the details and structure of the image. (3) Existing methods improve the performance by increasing the network depth or complexity, and also introduce a large number of redundant features, leading to an increase in network parameters and computational complexity. This not only makes the model training time longer, but also burdens the inference speed in practical applications.

To address these issues, we propose a dual-branch hybrid-scale feature aggregation network (HSFAN). By introducing a multi-scale parallel large convolution kernel module (MSPLCK) and a multi-scale large-kernel attention module (MSLA), HSFAN effectively fuses features across scales, breaking the single-scale information bottleneck and enabling cross-scale complementarity. Meanwhile, the enhanced parallel attention (EPA) module strengthens channel–spatial interactions, thereby capturing high-frequency details and edge textures more effectively and achieving efficient fusion of global semantics with local details. In addition, through a carefully designed architecture and optimized module layouts, HSFAN reduces redundant features while maintaining reconstruction quality, leading to a substantial decrease in computational complexity and parameter count. The main contributions of this work are as follows:

- We design a dual-branch feature extraction architecture: the main branch employs hybrid structural blocks to focus on local feature extraction, enhancing the recovery of high-information-entropy regions; the auxiliary branch combines multi-scale depthwise separable convolutions with dual attention mechanisms, effectively reducing computational complexity and strengthening global context modeling.
- We propose a Multi-Scale Parallel Convolution Module (MSPLCK). By using multiple group convolutions of different scales in parallel, it establishes a cross-scale information complementary mechanism, effectively capturing both large areas and fine textures in remote sensing images, and significantly enhancing the network’s capability to capture multi-scale features.
- We propose an Enhanced Parallel Attention (EPA) module that integrates multiple attention mechanisms in parallel to refine and filter feature streams, while extracting both globally shared information and position-dependent local cues, thereby enabling targeted enhancement and reconstruction of edge textures and high-frequency details in remote sensing images.
- We propose a Multi-Scale Large-Kernel Attention (MSLA) module that employs parallel depthwise separable convolutions to capture multi-level details while reducing computational overhead; it further integrates channel and spatial attention to strengthen salient features, thereby improving the model’s sensitivity to parcel boundaries in remote sensing images.

## 2. Related Works

### 2.1. Natural Image Super-Resolution Reconstruction Algorithm

In recent years, the CNN-based image super-resolution reconstruction task has been rapidly developing. Since Dong et al. [12] proposed the first CNN-based SR method, many related algorithms have been introduced, achieving superior performance. Some researchers seek to improve performance by increasing the network depth. For example, EDSR, proposed by Lim et al. [14], removes batch normalization in the traditional residual network and extends the number of convolutional layers to 50, significantly improving the reconstruction capability. The Residual Dense Network (RDN) designed by Zhang et al. [18] uses residual dense blocks to increase the network depth to 100 layers, capturing deeper image features. Additionally, Zhang et al. [19] designed the Residual Channel Attention Network (RCAN) with 400 layers, utilizing multiple dense residual connections to directly transmit low-frequency information and direct the model to focus on the reconstruction of high-frequency details. However, these methods generally neglect global dependencies while handling low-resolution features equally, resulting in inefficient recovery of image details. For this reason, researchers have attempted to introduce the attention mechanism into SR networks, aiming to guide the model to focus on key information and thus improve reconstruction performance. In this study, Guo et al. [20] proposed a Visual Attention Network (VAN), which efficiently achieves a wide range of receptive fields by combining deep convolution, dilated convolution, pointwise convolution layers, and the attention mechanism. Zhang et al. [21], on the other hand, integrated three attention modules into deep convolutional networks, solving the problems of feature underutilization and insufficient complementarity between channels and pixels. Although these methods have made progress in enhancing the global feature representation capability, the dense non-local modeling increases computational complexity and training costs. To improve computational efficiency and optimize user experience, researchers have begun exploring lightweight SR networks. In this study, Hui et al. [22] constructed the Information Multi-distillation Network (IMDN), which extracts multi-level features through a distillation mechanism, achieving a balance between reconstruction performance and memory consumption. The Efficient Super-Resolution Transformer (ESRT) proposed by Lu et al. [23] introduces an efficient multi-head attention mechanism that significantly reduces computational cost while enhancing the quality of image reconstruction. Wang et al. [24] proposed the Contextual Transformation Network (CTN), which reduces the number of parameters and optimizes network performance by lightweighting the convolutional layers. Wu et al. [25] introduced the Saliency-aware Dynamic Routing Network (SalDRN), which captures pixel-level features through a lightweight saliency detector, thereby reducing computational resources while maintaining high performance.

Parallel to the route focusing on architecture optimization, recent work has introduced contrastive learning into image restoration and enhancement, aligning the outputs through positive and negative constraints between the clear and degraded domains, thereby improving detail reconstruction and generalization ability in unpaired and cross-domain scenarios. For example, Wang et al. [26] designed a Content-Style Control Network with Style Contrastive Learning (CSC-SCL), which centers on content-style disentanglement. In the generator, it introduces Content-Controlling normalization (CCNorm) and Style-Controlling normalization (SCNorm) through frequency-domain amplitude and phase division to learn domain-invariant representations. Through style contrastive learning combined with content consistency constraints, the network aligns output styles with the clear domain while preserving semantic structures under unpaired and domain-label-free conditions, significantly enhancing the cross-domain generalization ability of underwater image enhancement methods. To alleviate model degradation caused by label noise in large-scale datasets, Zhang et al. [27] proposed a Progressive Sample Selection Framework with Contrastive Loss (PSSCL) for noisy labels. This framework first performs robust pretraining using Generalized Cross Entropy (GCE), and then combines Gaussian Mixture Models (GMM) with long-term confidence to select a small portion of high-confidence samples. In the subsequent semi-supervised learning phase, contrastive learning is introduced to progressively expand clean samples and enhance inter-class separability. Without requiring noise priors and with minimal hyperparameter adjustments, PSSCL achieves stable and leading robustness performance under various noise settings.

### 2.2. Remote Sensing Image Super-Resolution Reconstruction Algorithm

Remote sensing images [28,29] have important applications in fields such as geography and environmental monitoring. However, due to satellite and sensor limitations, super-resolution processing of low-resolution images is often required to obtain higher detail and accuracy. Lei et al. [30] proposed the Local–Global Combined Network (LGCNet), the first CNN-based super-resolution model for remote sensing images. It concatenates features at different levels through a “multi-branch” structure, effectively integrating local details and global environmental priors in remote sensing images. Subsequently, Haut et al. [31] proposed the DCM model, which improves super-resolution performance through multi-path feature fusion and lightweight enhancement while avoiding excessive network depth. Wang et al. [32] designed a Feature Enhancement Network (FeNet), utilizing a lightweight lattice block (LLB) for nonlinear feature extraction, and leveraging channel separation and attention mechanisms to promote efficient multi-branch interaction. In addition, some studies have introduced multi-scale modeling approaches to enhance feature representation capabilities. For example, Wang et al. [33] designed a Multi-scale Attention Network (MAN), which uses grouped convolution and dilated convolution for multi-scale feature modeling, successfully restoring multiple levels of features in remote sensing images. Lei et al. [34] proposed a Hybrid-scale Self-similarity Exploitation Network (HSENet), which uses a cross-scale connection structure (CCS) to capture similarities across different scales, further enhancing reconstruction performance.

In recent years, the Transformer [35] architecture has been widely applied to remote sensing image super-resolution reconstruction tasks. For example, Yoo et al. [36] introduced a cross-token attention mechanism within the Transformer framework, effectively exploiting information at different scales in remote sensing images. Sen et al. [37] constructed a Transformer-based enhancement network (TransENet), combining the multi-level enhancement structure of the Transformer with traditional SR frameworks to achieve effective fusion of multi-scale high- and low-dimensional features. Chen et al. [38] proposed a hybrid attention Transformer, enhancing interactions between neighboring window features by introducing an overlapping cross-attention module, thereby further improving the performance of the super-resolution model. Xiao et al. [39] proposed the Top-k Token Selective Transformer (TTST), which integrates multi-scale feature aggregation and global context attention mechanisms, significantly enhancing the fusion of global and local features. Guo et al. [40] integrated sub-pixel space mapping with sparse coding theory into the self-attention mechanism to construct a Sparse-activated Sub-pixel Transformer Network (SSTNet), enhancing the interactive optimization between convolutional and Transformer modules, significantly improving edge-texture fidelity in SR reconstruction.

## 3. Proposed Method

**Figure 1:**
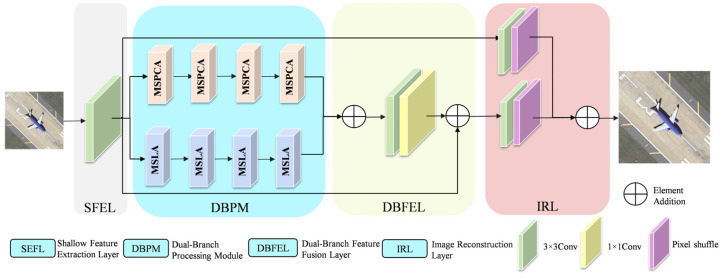
HSFAN overall architecture. Its core is a dual-branch structure composed of MSPCA and MSLA modules. The MSPCA module integrates MSPLCK and EPA, focusing on high-frequency feature extraction, while the MSLA module is responsible for multi-scale feature aggregation and key feature enhancement.

In this section, we first introduce the overall architecture of the HSFAN model, as shown in Figure 1. Subsequently, we elaborate three key improvements in the model, specifically: the multi-scale parallel large convolutional kernel (MSPLCK) module for enhancing the ability to capture image edge details and high-frequency information, the enhanced parallel attention (EPA) module for dealing with complex structural details and inhomogeneous degradation, and the multi-scale large kernel attention (MSLA) module for enhancing the multi-scale feature representation. Finally, we introduce the loss function used in the method of this paper.

### 3.1. Overall Model Structure

The overall structure of the HSFAN consists of four parts: a shallow feature extraction layer, a dual-branch processing module, a dual-branch feature fusion layer, and an image reconstruction layer. Firstly, the shallow feature extraction layer extracts shallow features $F_{SEFL}$ from a given low-resolution image $I_{LR}$ through a 3 × 3 convolutional layer, captures the basic edge information and local details of the image, and provides preliminary feature representation for subsequent processing. The specific formula is:

(1) $$F_{SFEL}={Conv}_{3\times 3}\left(I_{LR}\right).$$

where $F_{SFEL}$ represents the extracted shallow features, and ${Conv}_{3\times 3}$ denotes a 3 × 3 convolutional layer. The extracted shallow features are passed to subsequent layers and directly input into the upsampling module to provide low-frequency information for image reconstruction.

The dual-branch processing module consists of four Multi-Scale Parallel Convolutional Attention (MSPCA) modules and four MSLA modules. Among them, MSPCA, composed of MSPLCK and EPA, is mainly responsible for extracting global feature information and, through the synergy of multi-scale convolutions and attention mechanisms, enhancing the network’s ability to perceive and capture key spatial information—thereby better capturing important details and structural features in the image. The MSLA module, serving as the auxiliary branch, fuses multi-scale features from different stages via parallel multi-scale depthwise separable convolutions, effectively optimizing information extraction and feature fusion, and strengthening the network’s ability to capture spatial and channel information as well as complex patterns. The extraction of deep features from the shallow features $F_{SEFL}$ through four dual-branch processing modules can be expressed as:

(2) $$F_{i}=C_{MSPCA}^{i}\left(F_{i}\right)⊕C_{MSLA}^{i}\left(F_{i}\right),1\leq i\leq 4.$$

where $F_{i}$ represents the fused features extracted at the i−th MSPCA block $C_{MSPCA}^{i}$ and the i−th MSLA block $C_{MSLA}^{i}$. Subsequently, the deep features extracted by the MSPCA and MSLA modules are input into the dual-branch feature fusion layer through residual connections, resulting in the fused feature $F_{DBFE}$. This method achieves efficient feature fusion without increasing network parameters, as expressed by:

(3) $$F_{DBFE}={Conv}_{3\times 3}\left({Conv}_{1\times 1}\left(F_{MSPCA}^{Concat}⊕F_{MSLA}^{Concat}\right)\right)+F_{SFEL}.$$

Finally, the fusion feature $F_{DBFE}$ and the initial shallow extraction feature $F_{SEFL}$ are upsampled by sub-pixel convolutional processing to generate the final super-resolution remote sensing image. The expression is as follows:

(4) $$I_{SR}=H_{UP}\left({Conv}_{3\times 3}\left(F_{DBFE}\right)+{Conv}_{3\times 3}\left(F_{SEFL}\right)\right).$$

where $H_{UP}$ represents the Pixel Shuffle operation, which increases the image resolution by rearranging the pixels of the low-resolution feature map.

### 3.2. Multi-Scale Parallel Large Convolution Kernel Module

**Figure 2:**
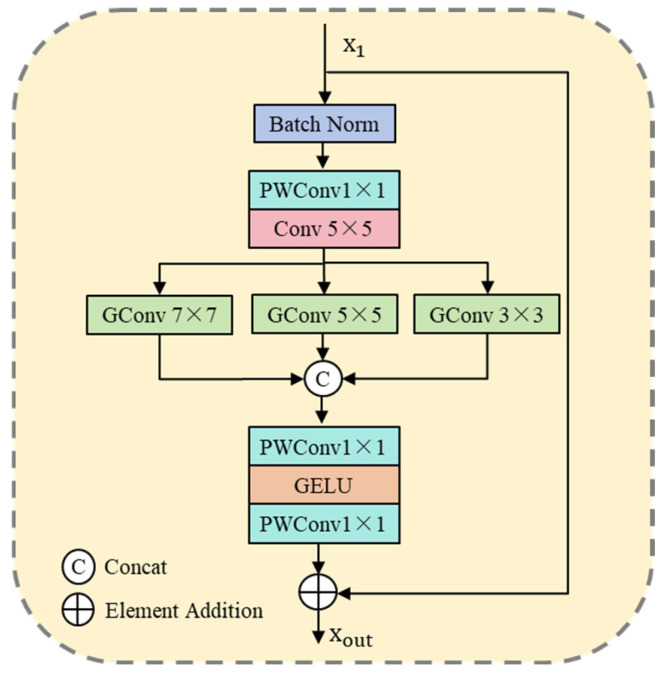
The MSPLCK simulates multi-scale receptive fields by applying convolution kernels of different sizes in parallel, establishing a cross-scale information complement mechanism, and thus capturing multi-granularity information flow from global to local.

MSPLCK applies convolution kernels of different sizes in parallel to emulate receptive fields at multiple scales, thereby establishing a cross-scale information complementarity mechanism. By treating each kernel as a parallel information-processing channel, it collaboratively captures multi-granularity signals in remote sensing images—from global context to local details—enabling more comprehensive and efficient encoding of edge structures and high-frequency content. Figure 2 shows its basic structure.

The input feature map $x_{1}$ is first normalised through the Batch Normalisation layer to speed up the training and improve the stability of the model. Subsequently, the processed features are prepared for subsequent convolutions by adjusting the number of channels through Point-Wise Convolution (PWC), and then convolution operations are performed using 5 × 5 convolution kernels to capture the mesoscale features of the image. The expression is as follows:

(5) $$x_{2}={Conv}_{5\times 5}\left({PWConv}_{1\times 1}\left(x_{1}\right)\right).$$

where ${PWConv}_{1\times 1}$ denotes point-wise convolution with a 1 × 1 convolution kernel, and $x_{2}$ represents the output feature map after the application of pointwise convolution and regular convolution. Subsequently, the output feature $x_{2}$ will be passed into three group convolutions (GC) with different kernel sizes in parallel to further capture the features at different scales. In these grouped convolutions, the large convolution uses the 7 × 7 convolutional kernel to extract global features and identify and process a wide range of primary features of remote sensing images. In the medium convolution, the 5 × 5 convolution kernel is used to understand the moderately complex structural features of the image, balancing the receptive field and computational efficiency. The small convolution, on the other hand, uses a 3 × 3 convolutional kernel to focus on the high-frequency region of the remote sensing image to further restore the image details and local features in detail. The expression is as follows:

(6) $$\left\{\begin{array}{c} f_{large}={GConv}_{7\times 7}\left(x_{2}\right),\\ f_{medium}={GConv}_{5\times 5}\left(x_{2}\right),\\ f_{small}={GConv}_{3\times 3}\left(x_{2}\right). \end{array}\right.$$

where ${GConv}_{k\times k}$ denotes a grouped convolution operation using k×k convolution kernels, and k can be 3, 5, or 7, corresponding to small, medium, and large convolution kernels, respectively. Then, the information from different scales is connected in the channel and feature dimensions and input into a multi-layer perceptron (MLP), which can convert the dimensions of the input features into the same dimensions $x_{1}$ as the original input feature map, and add the output with the original input features through residual joining, so as to promote information flow and gradient propagation, and improve the efficiency of network training. The expression is as follows:

(7) $$x_{out}=x_{1}+MLP\left(Concat(f_{large},f_{medium},f_{small})\right).$$

where $x_{out}$ represents the final output feature map, obtained by adding the original input features and the features processed by the MLP through a residual connection. Concat(⋅) refers to the operation of concatenating the output feature maps of different group convolutions along the channel dimension.

### 3.3. Enhanced Parallel Attention

**Figure 3:**
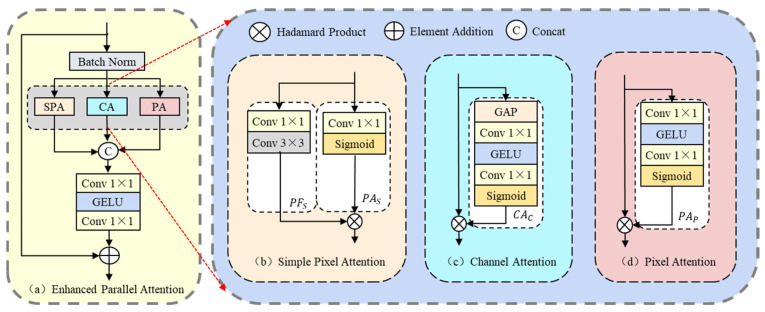
(**a**) Overall structure of the enhanced parallel attention (EPA) module. (**b**) Simple pixel attention (SPA). (**c**) Channel attention (CA). (**d**) Pixel attention (PA). The red dashed arrows indicate the correspondence between the EPA submodules in (**a**) and their detailed structures in (**b**–**d**).

The EPA integrates multiple attention mechanisms in parallel, explicitly strengthening channel–spatial interactions and multi-scale information fusion; without a significant increase in overhead, it balances global semantics and local details, suppresses redundancy, and efficiently handles complex structures and non-uniform degradations, thereby improving the model’s performance and adaptability. Its basic structure is shown in Figure 3.

The initial feature map $x_{1}$ is normalised through the Batch Normalisation layer, and then passed in parallel to the Simple Pixel Attention (SPA), Channel Attention (CA) and Pixel Attention (PA) modules. The simple pixel attention module is used to extract position-dependent local features, which contains a feature extraction branching $PF_{S}$ and a pixel gate branching $PA_{S}$, in which $PA_{S}$ is used as a $PF_{S}$ pixel gating signal. The two are fused by multiplying each element for information. The structure is shown in Figure 3b, and the specific expression is:

(8) $$PF_{S}={Conv}_{3\times 3}\left({PWConv}_{1\times 1}\left(x_{1}\right)\right).$$

(9) $${PA}_{S}=Sigmoid\left({PWConv}_{1\times 1}\left(x_{1}\right)\right).$$

(10) $$F_{S}=PF_{S}⊗PA_{S}.$$

where $F_{S}$ represents the feature map processed by the Simple Pixel Attention module. ${PWConv}_{1\times 1}$ denotes pointwise convolution, and ${Conv}_{3\times 3}$ refers to convolution with a kernel size of 3. Pixel attention uses the global perception mechanism to capture the important information of each pixel, generate pixel-level gating signals, enhance the reconstruction effect of key areas, and suppress irrelevant information. It extracts global pixel-level features through the ${PA}_{S}$ branch; the basic structure is shown in Figure 3b, and the expression is:

(11) $$PA_{P}=Sigmoid\left({PWConv}_{1\times 1}\left(GELU\left({PWConv}_{1\times 1}(x_{1})\right)\right)\right).$$

(12) $$F_{p}=x_{1}⊗{PA}_{P}.$$

where $F_{p}$ represents the feature map processed by the pixel attention module. The Sigmoid function is used to extract global pixel-gated features. ${PA}_{P}$ is represented as a global pixel gating signal of $x_{1}$. Channel attention captures global information between channels through global average pooling, and updates each channel of the feature map by multiplying each element. Typically, the entire channel feature is extracted using the ${CA}_{c}$ branch, the basic structure of which is shown in Figure 3d with the following expression:

(13) $${CA}_{c}=Sigmoid\left({PWConv}_{1\times 1}\left(GELU\left({PWConv}_{1\times 1}\left(GAP\left(x_{1}\right)\right)\right)\right)\right).$$

(14) $$F_{C}=x_{1}⊗{CA}_{c}.$$

where $F_{C}$ represents the feature map processed by the channel attention module. GAP stands for Global Average Pooling, and ${CA}_{c}$ is the global channel gating signal for $x_{1}$. The output feature map processed by SPA, CA and PA mechanisms will be stitched in the channel dimension, and then processed by MLP and then connected with the input features by residual to obtain the final output feature map. The specific expression is:

(15) $$F_{final}=Concat\left(F_{S},F_{c},F_{P}\right).$$

(16) $$y=x_{1}+{PWConv}_{1\times 1}\left(GELU\left({PWConv}_{1\times 1}\left(F_{final}\right)\right)\right).$$

where $F_{final}$ represents the output feature map obtained by splicing $F_{S}$, $F_{c}$, and $F_{P}$. Concat(⋅) represents the operation of stitching multiple feature maps along the channel dimension. y is the output feature map processed by MLP, which is connected with the residual $x_{1}$ input feature map.

### 3.4. Multi-Scale Large-Kernel Attention

**Figure 4:**
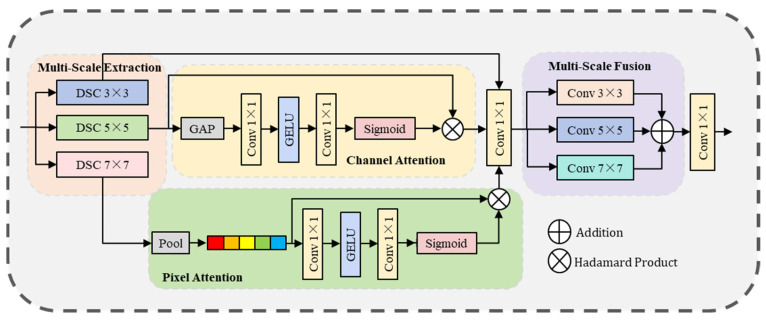
The MSLA module sequentially performs preliminary feature extraction using multi-scale depthwise separable convolutions, enhances features with a dual-attention mechanism, and finally refines fine-grained features via multi-scale convolutions.

The MSLA module extracts features at different stages by parallel use of multi-scale depthwise separable convolutions, constructing a multi-path information complementarity mechanism. This design not only enhances multi-scale information and spatial–channel interaction, but also significantly improves feature encoding efficiency through depthwise separable convolutions, thereby strengthening feature representation in both spatial and channel dimensions. This is crucial for preserving the continuity of land structures and high-frequency details in large-scale remote sensing images, effectively alleviating information loss caused by single-scale feature extraction. Figure 4 illustrates its basic structure.

The input feature $x_{1}$ is first passed through three parallel depth-separable convolutions (3 × 3, 5 × 5, 7 × 7) to extract features at multiple scales. The specific expression is:

(17) $$\left\{\begin{array}{c} x_{3}={DSConv}_{3\times 3}\left(x_{1}\right),\\ x_{5}={DSConv}_{5\times 5}\left(x_{1}\right),\\ x_{7}={DSConv}_{7\times 7}\left(x_{1}\right). \end{array}\right.$$

where DSConv stands for depth separable convolutional. Among them, all convolutions with padding keep the resolution unchanged. Subsequently, the channel attention module was applied to the extracted feature map $x_{5}$ to adaptively adjust the channel weights to strengthen the important features. For feature map $x_{7}$, the spatial dimension is compressed to 1 × 1, the global information is extracted through maximum pooling and average pooling, and then the information is processed using 1 × 1 convolution and nonlinear transformation is introduced through GELU activation. After that, the Sigmoid activation function is used to generate a spatial attention mechanism. Finally, the processed feature maps $x_{3}$, $x_{5}$ and $x_{7}$ were stitched together and fused feature $F_{proj}$ was obtained by projection processing.

(18) $$CA=\sigma ({Conv}_{1\times 1}\left(ReLU({Conv}_{1\times 1}(GAP\left(x_{5}\right))))\right).$$

(19) $$SA=\sigma ({Conv}_{7\times 7}\left(Concat\left(Maxpool,AvgPool\right))\right).$$

(20) $$F_{proj}={Conv}_{1\times 1}\left(Concat\left(x_{3},x_{5},x_{7}\right)\right).$$

where σ represents the Sigmoid activation function. The features extracted by multi-scale depth separable convolution are spliced in the channel dimension to obtain the dimensionally reduced feature $x_{down}$. Subsequently, the $x_{down}$ captures multi-scale spatial features through ordinary convolutional convolutions of three different kernel sizes (3 × 3, 5 × 5, 7 × 7) to generate three feature maps containing information ranging from fine-grained to coarse-grained. Finally, the final enhanced feature map $F_{enhanced}$ was obtained by average fusion of the three feature maps and the ascending dimension of the 1 × 1 convolutional layer. This process not only enhances the ability to express features, but also improves the sensitivity and recognition ability of the model to features at different scales. The specific expression is as follows:

(21) $$x_{down}={Conv}_{1\times 1}\left(Concat\left(x_{3},x_{5},x_{7}\right)\right).$$

(22) $$f=\left[{Conv}_{3\times 3}\left(x_{down}\right),{Conv}_{5\times 5}\left(x_{down}\right),{Conv}_{7\times 7}\left(x_{down}\right)\right].$$

(23) $$F_{enhanced}={Conv}_{1\times 1}\left(\frac{f}{3}\right).$$

where f represents a vector containing three feature maps, each of which is the result of convolution of different kernel sizes.

### 3.5. Loss Function

In this paper, the L1 loss function between the super-resolution image $I_{SR}$ and the ground truth high-resolution image $I_{HR}$ is used to guide the network training. This loss function measures the pixel-wise difference between the predicted and ground truth images and optimizes the model by minimizing this difference. The computation is expressed as follows:

(24) $$L_{SR}={\left\|I_{SR}-I_{HR}\right\|}_{1}.$$

where $\left\|\cdot \right\|$ denotes the L1 norm.

## 4. Experiments and Analysis

### 4.1. Datasets

**Figure 5:**
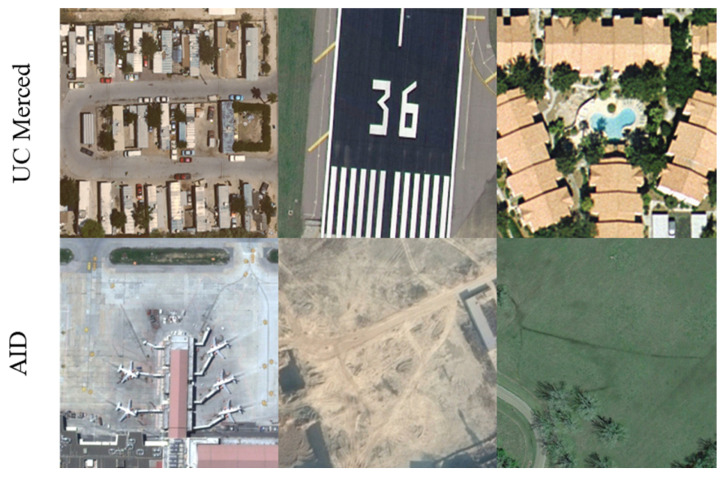
Several representative image samples from the AID and UC Merced datasets.

To evaluate the effectiveness of the proposed algorithm, we conducted extensive experiments on the AID [41] and UC Merced [42] datasets. The AID dataset comprises 10,000 remote sensing images across 30 categories (e.g., deserts, beaches, airports), with 220–420 images per category and a resolution of 600 × 600 pixels. For this study, 8000 images were randomly sampled for training and 2000 for testing (an 8:2 split); in addition, 150 images (5 per category) were randomly selected as a validation set. The UC Merced dataset contains 2100 RGB images of 256 × 256 pixels spanning 21 categories (e.g., roads, farmland, shrubs). We used 1050 images for training and the remaining 1050 for testing; furthermore, 10% of the test set was randomly selected for validation. Figure 5 presents representative samples from the AID and UC Merced datasets.

### 4.2. Experimental Parameters

This paper focuses on experimental research with scaling factors of ×2, ×3 and ×4 for remote sensing image data. The experiments first degrade HR images using bicubic downsampling to generate LR images, creating HR-LR image pairs. In each training iteration, the network inputs 16 low-resolution image patches of size 48 × 48 and uses horizontal flipping and random rotations for data augmentation. The Adam optimizer is employed for model training, with parameters set as $\beta_{1}=0.9$ and $\beta_{2}=0.99$. The initial learning rate is set to $5\times 10^{-4}$, halved every 400 epochs, with a total of 800 epochs. The batch size is set to 16. To prevent overfitting, early stopping is introduced with a patience of 500 epochs, and the L1 loss function is used. The experiments are conducted within the PyTorch framework, utilizing Python 3.9.19 and CUDA 12.4, with training and testing performed on a workstation running Microsoft Windows 10 (Microsoft, Raymond, DC, USA), equipped with an Intel Core i7-12700KF CPU (Intel, Santa Clara, CA, USA), 32 GB of RAM, and a Gigabyte graphics card (Gigabyte Technology, Taipei, Taiwan) based on the NVIDIA GeForce RTX 4060 Ti 16 GB GPU (NVIDIA, Santa Clara, CA, USA).

### 4.3. Evaluation Metrics

In this paper, Peak-Signal-to-noise ratio (PSNR) [43] and Structural Similarity (SSIM) [44] are used for objective evaluation. PSNR measures the distortion level of an image by calculating the error in dB between the reconstructed HR image and the corresponding pixel of the real HR image. The PSNR measures the degree of distortion in dB, which is mainly determined by the mean square error (MSE). SSIM is a metric used to measure the similarity between two images, which usually depends on the brightness, contrast and structural information of the image. The expression for both is:

(25) $$PSNR=10\times {log}_{10}\left(\frac{{MAX}^{2}}{MSE}\right).$$

(26) $$SSIM\left(x,y\right)=\frac{\left(2\mu_{x}\mu_{y}+c_{1}\right)\left(2\sigma_{xy}+c_{2}\right)}{\left(\mu_{x}^{2}+\mu_{y}^{2}+c_{1}\right)\left(\sigma_{x}^{2}+\sigma_{y}^{2}+c_{2}\right)}.$$

where MAX denotes the maximum pixel value in the reference image, with a value of 255. σ denotes the standard deviation, μ denotes the mean value, and $c_{1}$, $c_{2}$ are constants usually used for stabilization calculation. PSNR takes the value of [0, +∞), and the larger the value is, the smaller the pixel error; the smaller the degree of image distortion between the reconstructed HR image and the real HR image is, the higher the quality of reconstruction. SSIM takes the value of [0, 1], and the closer to 1 the value of SSIM is, the better the quality of the reconstructed image.

### 4.4. Comparative Experiments

In order to verify the effectiveness of the proposed algorithm, this paper conducts a comparative analysis with a variety of advanced super-resolution algorithms, including Bicubic, SRCNN [12], LGCNet [30], IMDN [22], CTNet [24], ESRT [23], DCM [31], FeNet [32], ACT [36], TransEnet [37], and SSTNet [40], through three aspects: objective evaluation index, subjective visual effect and model complexity. The experiment was performed on the UC Merced and AID datasets, and the images at ×2, ×3 and ×4 magnifications were super-reconstructed, respectively. The specific experimental results are shown in Table 1.

#### 4.4.1. Objective Evaluation Index Comparison

From the quantitative super-resolution results (×2, ×3, ×4) on the UC Merced and AID datasets shown in Table 1, it can be seen that our algorithm demonstrates good reconstruction performance across different datasets and scaling factors. Specifically, on the UC Merced dataset, compared with lightweight models such as LGCNet, IMDN, and FeNet, our method has an advantage in reconstruction quality. Taking the DCM model as an example, for the ×2 and ×3 tasks, our algorithm achieves PSNR improvements of 0.42 dB and 0.45 dB, and SSIM increases of 0.0036 and 0.0013, while the number of parameters is reduced by 0.31 M and 0.10 M, respectively. In the ×4 SR task, further comparisons with high-performance models such as ACT, TransEnet, and SSTNet show that our model achieves PSNR improvements of 0.37 dB, 0.10 dB, and 0.25 dB, respectively, while its number of parameters is only 5.1%, 6.3%, and 6.9% of those models. This fully demonstrates that the proposed method can significantly reduce learnable parameters while attaining performance gains.

Experiments on the AID dataset further validate the advantages of our algorithm. Compared with lightweight models CTNet, ESRT, and FeNet, our method improves the PSNR of reconstructed images by 0.20 dB, 0.18 dB, and 0.18 dB in the ×2 upscaling task, respectively. In the more challenging ×4 task, our algorithm still maintains a significant advantage over the high-performance models ACT, TransEnet, and SSTNet, with PSNR improvements of 0.33 dB, 0.14 dB, and 0.18 dB, respectively. Although the performance is similar to TransEnet and SSTNet at the ×3 scale, the number of parameters of our model is significantly reduced. These results are attributed to the effective fusion of multi-scale information by the proposed network and the coordinated enhancement of local and global structures in remote sensing images.

Moreover, to further verify the effectiveness of the proposed algorithm, this paper compares 21 scene categories with other advanced algorithms at three scales of the UC Merced dataset. Table 2 presents the specific comparison results, where bold numbers indicate the best results and underlined numbers indicate the second-best results. The experimental results show that the proposed algorithm achieves the highest PSNR in 11 scenes and exhibits relatively small PSNR fluctuations across different categories. These results fully demonstrate that the proposed algorithm has better stability and stronger scene adaptability for different scenarios.

#### 4.4.2. Subjective Comparison

**Figure 6:**
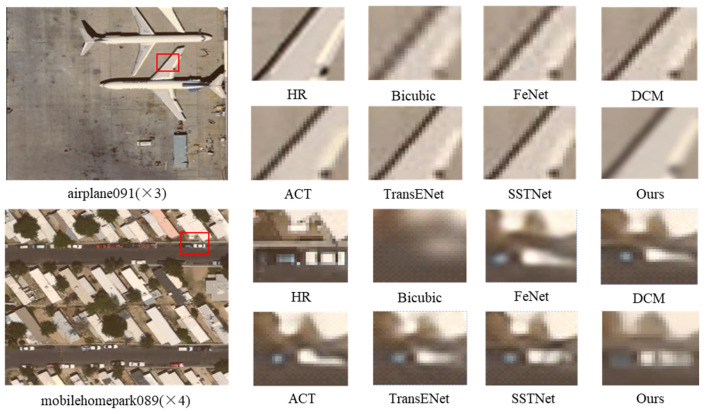
Subjective visualization of different methods on the UC Merced dataset. The red boxes indicate the regions that are enlarged in the subsequent columns.

To further verify the effectiveness of the proposed algorithm and evaluate its visual quality compared to current leading methods, experiments were conducted on the ×3 and ×4 scales of the UC Merced and AID datasets to compare the reconstructed images of different methods. As shown in Figure 6, at the ×3 magnification scale, the enlarged local details of the airplane091.tif image reveal that the images reconstructed by Bicubic and FeNet are blurry and lack edge details, while the images reconstructed by DCM, ACT, and TransENet exhibit jagged edges. In contrast, the wing edges reconstructed by the proposed algorithm are sharper with clearer textures. For the mobilehomepark089.tif image at the ×4 magnification scale, except for the Bicubic method, other methods can restore the main outline of the image but show varying degrees of distortion, whereas the image reconstructed by our algorithm appears clearer and more natural.

**Figure 7:**
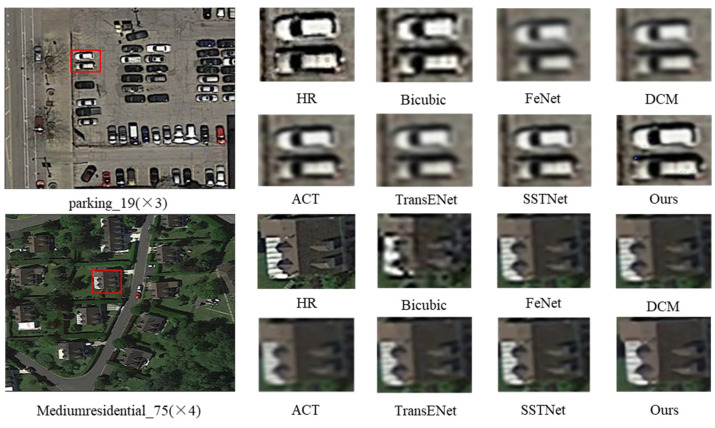
Subjective visualization of different methods on AID dataset. The red boxes indicate the regions that are enlarged in the subsequent columns.

Figure 7 illustrates the comparison of reconstructed images by different methods on the AID dataset. At the ×3 magnification scale of the parking_19 image, the Bicubic method produces noticeable blur, while FeNet, DCM, and TransENet recover better details, with our algorithm preserving the vehicle’s edge contours more effectively and providing higher clarity. At the ×4 magnification scale of the Mediumresidential_75 image, our algorithm outperforms other methods in the details of buildings and trees, offering clearer details and less noise compared to the HR image.

#### 4.4.3. Model Complexity Analysis

**Figure 8:**
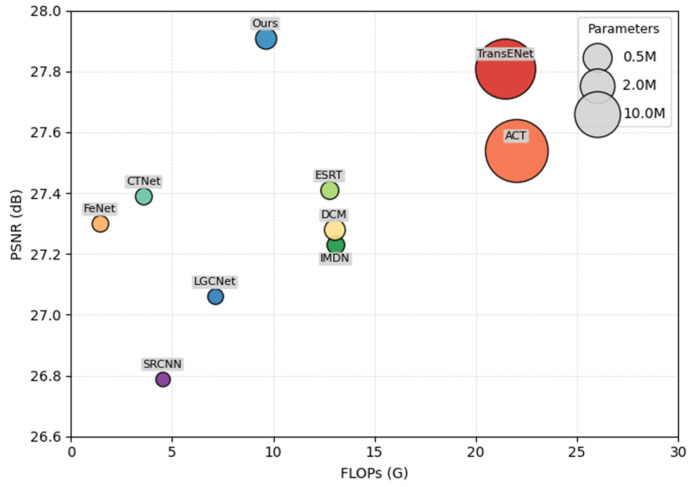
Comparison of performance and complexity of different methods.

Table 3 shows the performance and complexity analysis of the model proposed in this paper compared with other advanced methods on the UC Merced dataset at a ×4 upscaling factor. Here, FLOPs represent the number of floating-point operations, reflecting the computational complexity of the model, while the number of parameters measures the storage requirements. By comparing the FLOPs and parameter counts of different methods, the efficiency and effectiveness of our model in practical applications can be more comprehensively evaluated. For easier comparison, we plotted the performance and complexity comparison of different models in Figure 8. Compared with lightweight models such as ESRT, FeNet, and IMDN, the algorithm proposed in this paper achieves a better balance between image reconstruction quality and computational efficiency, with performance significantly superior to these methods. Compared with high-performance models such as TransENet and ACT, our algorithm can maintain high performance while having lower computational complexity and storage requirements.

In summary, at ×4 upscaling, HSFAN achieves the highest PSNR (27.91 dB) with only 9.60 GFLOPs and 2.37 M parameters. In terms of performance, HSFAN’s PSNR is 0.50 dB, 0.61 dB, and 0.68 dB higher than lightweight models such as ESRT, FeNet, and IMDN, respectively. In terms of computational complexity, its requirements are less than 45% of those of TransENet (21.44 GFLOPs) and ACT (22.00 GFLOPs). These results suggest that the performance improvement is primarily attributed to the three key components proposed, which effectively capture cross-scale information and long-range dependencies between similar image patches, rather than simply increasing network depth or computational cost.

### 4.5. Ablation Experiments

To comprehensively evaluate the HSFAN model, we conducted ablation experiments on its various components. To improve experimental efficiency, all ablation studies were performed on the UC Merced dataset with a ×4 magnification factor. Each set of experiments was trained for 500 epochs, and the experimental setup and test set division remained consistent with the previously mentioned settings.

#### 4.5.1. Effectiveness Ablation Analysis of Each Component

To validate the effectiveness of the proposed algorithm, experiments were conducted by removing each module one by one to observe the performance changes, thereby assessing the contribution of each module. Table 4 shows that the baseline model performed the worst after all three modules were removed, with a PSNR of 27.44; whereas when all three modules worked together, the super-resolution reconstruction achieved the best results, with PSNR and SSIM values of 27.91 and 0.7696, respectively. After removing the MSPLCK module, the loss of single-scale feature processing resulted in the loss of edge texture details in remote sensing images, causing performance degradation. After removing the EPA module, the absence of the parallel attention mechanism prevented the model from focusing on key feature regions, weakening the global feature fusion ability and affecting the reconstruction performance. When the MSLA module was removed, the reduced correlation of local region features led to performance decline. Furthermore, when the dual-branch structure used only any one of the MSPLCK, EPA, or MSLA modules, model performance was significantly reduced. In summary, after combining all the proposed modules, the overall network performance was significantly improved, thereby demonstrating the effectiveness of the proposed modules.

#### 4.5.2. Effectiveness Analysis of the MSPLCK Module

To validate the innovation of the Multi-Scale Parallel Convolution Module (MSPLCK) and its advantages in capturing multi-scale features and expanding the receptive field, three sets of comparative experiments were designed, employing parallel convolutional kernels of identical scales for testing. Firstly, the multi-scale grouped convolutions within the MSPLCK module were replaced with three parallel convolutional kernels of the same scale (${GConv}_{3\times 3}$, ${GConv}_{5\times 5}$, ${GConv}_{7\times 7}$). The experimental results are presented in Table 5. When employing parallel convolutional kernels of identical scales, the model’s PSNR and SSIM metrics exhibited a marked decline. Conversely, the concurrent utilization of convolutional kernels at varying scales enabled effective capture of information across multiple scales, leading to a significant performance enhancement. This thoroughly validates the pivotal role of multi-scale parallel convolutional kernels in improving the quality of detail recovery and reconstruction in remote sensing images, demonstrating our method’s distinctive advantages in feature extraction.

#### 4.5.3. Effectiveness Analysis of the EPA Module

To validate the advantages of parallel attention mechanisms in enhancing edge textures and high-frequency features in remote sensing images, three comparative experiments were conducted to evaluate the performance of serial versus parallel attention. First, the three parallel attention mechanisms within the EPA module were replaced with serial CA and PA mechanisms. Subsequently, the three parallel attention mechanisms were substituted with serial SPA, CA, and PA mechanisms. The experimental results are presented in Table 6. The findings indicate that simply stacking different attention mechanisms did not enhance model performance; rather, it led to a decline in performance and increased computational complexity. Conversely, by adopting a parallel architecture design, the model achieved a significant performance boost with only a marginal increase in parameter count and computational complexity. This outcome fully validates the effectiveness and innovative nature of our approach.

#### 4.5.4. Effectiveness Analysis of the MSLA Module

To validate the effectiveness of the Multi-Scale Large-Kernel Attention (MSLA) module in a dual-branch architecture and its sensitivity to remote sensing image information, four controlled experiments were conducted. M9 replaces the MSLA module with conventional convolution (${Conv}_{3\times 3}$); M10 removes the multi-scale depthwise separable convolution; M11 excludes the channel and spatial attention mechanisms; and M12 represents the complete model. The results, shown in Table 7, reveal that substituting the MSLA module with conventional convolution (M9) increases model complexity and reduces performance, indicating that conventional convolution cannot effectively capture image details. Removing the multi-scale depthwise separable convolution (M10) further degrades performance, highlighting the importance of multi-scale feature integration. Moreover, excluding the attention mechanisms (M11) leads to lower performance than the complete model (M12), confirming the vital role of dual attention in enhancing sensitivity to image details. These findings demonstrate that combining multi-scale depthwise separable convolution with dual-attention mechanisms significantly improves performance while maintaining low computational complexity, showcasing the effectiveness and innovation of the MSLA module in enhancing detail capture and information sensitivity in remote sensing images.

## 5. Conclusions

This paper proposes a remote sensing image super-resolution network based on a dual-branch hybrid-scale feature aggregation architecture. By employing a multi-scale parallel large convolution kernel module, the network captures both large-area regions and detailed textures, establishing a cross-scale information complementarity mechanism that significantly expands the model’s receptive field and enhances the representation of image details. Meanwhile, to better handle the edges and textures in complex remote sensing images, multiple attention mechanisms are applied in parallel to optimize and filter the feature flow, extracting both global consistency and local region information, and adaptively enhancing high-information-entropy features. Additionally, a dual-branch structure is constructed by integrating the multi-scale large-kernel attention module, further optimizing detail extraction and feature enhancement, thus improving the model’s ability to recover spatial details and its reconstruction performance. Experimental results demonstrate that, on the AID and UC Merced datasets, the proposed method outperforms existing state-of-the-art algorithms in terms of PSNR and SSIM, while also reducing computational complexity and storage requirements without sacrificing performance. Ablation experiments further validate the effectiveness of each module, confirming the rationality and innovation of the overall design, and providing an efficient and robust solution for remote sensing image super-resolution tasks.

Although the proposed method strikes a good balance between performance and efficiency, the current model structure, including the parallel attention mechanism and multi-branch fusion strategy, still contains some redundancy in terms of parameters and computational complexity. Future work will focus on optimizing the model’s real-time performance, enhancing its adaptability in complex remote sensing scenarios, and exploring more streamlined and efficient network architectures. Furthermore, further theoretical analysis, including rate-distortion tradeoff analysis from information theory, will provide deeper insights for optimizing the design, which is an important direction for future research.

## Figures and Tables

**Table 1:** Comparison of the average PSNR (dB)/SSIM performance of different models on the UC Merced and AID datasets for ×2, ×3, and ×4 SR (the best results are highlighted in red).

Method	Scale	Params	UC Merced		AID	
			PSNR/dB	SSIM	PSNR/dB	SSIM
Bicubic	×2	-/-	30.76	0.8789	32.39	0.8906
SRCNN [12]		57 K	32.86	0.9077	34.49	0.9286
LGCNet [30]		193 K	33.73	0.9231	35.05	0.9312
IMDN [22]		694 K	33.52	0.9243	35.10	0.9346
CTNet [24]		413 K	33.61	0.9264	35.13	0.9354
ESRT [23]		750 K	33.70	0.9270	35.15	0.9358
DCM [31]		2.18 M	33.68	0.9277	35.21	0.9366
FeNet [32]		351 K	33.62	0.9264	35.15	0.9361
ACT [36]		46.1 M	33.88	0.9283	35.17	0.9362
TransEnet [37]		37.3 M	34.06	0.9294	35.28	0.9374
SSTNet [40]		34.14 M	34.09	0.9311	35.29	0.9376
Ours		1.87 M	34.10	0.9313	35.33	0.9376
Bicubic	×3	-/-	27.46	0.7631	29.08	0.7863
SRCNN [12]		57 K	28.92	0.8116	30.60	0.8380
LGCNet [30]		193 K	29.29	0.8241	30.73	0.8417
IMDN [22]		694 K	29.42	0.8315	31.14	0.8518
CTNet [24]		413 K	29.41	0.8322	31.16	0.8527
ESRT [23]		750 K	29.52	0.8318	31.23	0.8527
DCM [31]		2.18 M	29.52	0.8391	31.31	0.8561
FeNet [32]		351 K	29.47	0.8320	31.25	0.8538
ACT [36]		46.1 M	29.80	0.8395	31.39	0.8576
TransEnet [37]		37.3 M	29.92	0.8406	31.45	0.8585
SSTNet [40]		34.14 M	29.88	0.8397	31.45	0.8595
Ours		2.08 M	29.97	0.8404	31.56	0.8577
Bicubic	×4	-/-	25.65	0.6725	27.30	0.7036
SRCNN [12]		57 K	26.79	0.7243	28.45	0.7560
LGCNet [30]		193 K	27.06	0.7339	28.61	0.7632
IMDN [22]		694 K	27.23	0.7432	28.96	0.7698
CTNet [24]		413 K	27.39	0.7513	29.00	0.7768
ESRT [23]		750 K	27.41	0.7485	29.18	0.7831
DCM [31]		2.18 M	27.28	0.7533	29.17	0.7824
FeNet [32]		351 K	27.30	0.7447	29.08	0.7804
ACT [36]		46.1 M	27.54	0.7531	29.19	0.7836
TransEnet [37]		37.3 M	27.81	0.7639	29.38	0.7909
SSTNet [40]		34.14 M	27.66	0.7598	29.34	0.7896
Ours		2.37 M	27.91	0.7696	29.52	0.7928

**Table 2:** PSNR (/dB) of each category under scale factor ×3 on the UC Merced dataset. The best results are highlighted in bold, and the second best are underlined.

Class	SRCNN	LGCNet	IMDN	CTNet	ESRT	FeNet	DCM	ACT	TransEnet	SSTNet	Ours
Agricultural	27.44	27.64	28.49	28.53	28.13	28.45	**29.06**	27.87	28.02	28.02	28.50
Airplane	28.65	29.23	29.23	29.22	29.45	29.35	30.66	29.78	29.94	29.94	**30.67**
Baseballdiamond	34.56	34.75	34.95	34.81	34.88	34.81	33.87	35.03	**35.04**	**35.04**	**35.04**
Beach	37.11	37.28	37.46	37.38	37.45	37.46	36.38	37.56	37.53	**37.59**	37.27
Buildings	27.22	27.88	27.97	27.99	28.18	28.10	28.51	28.66	28.81	28.74	**29.15**
Chaparral	26.18	26.35	26.42	26.40	26.43	26.39	26.81	26.62	26.69	26.69	**26.88**
Denseresidential	27.77	28.29	28.43	28.42	28.53	28.42	28.79	28.97	29.11	**29.12**	28.45
Forest	28.35	28.41	28.42	28.48	28.47	28.47	28.16	28.56	28.55	28.57	**29.05**
Freeway	28.89	29.55	29.54	29.60	29.87	29.75	30.45	30.25	30.38	30.47	**30.57**
Golfcourse	36.33	36.46	36.47	36.46	36.54	36.49	34.43	36.63	**36.68**	36.65	36.44
Harbor	23.09	23.61	23.84	23.83	23.87	23.77	25.55	24.42	24.72	24.56	**25.66**
Intersection	27.91	28.32	28.40	28.38	28.53	28.47	**29.28**	28.85	29.03	29.02	29.15
Mediumresidential	27.35	27.78	27.83	27.87	27.93	27.89	27.77	28.30	**28.47**	28.42	27.85
Mobilehomepark	24.23	24.70	24.90	24.87	24.92	24.88	24.94	25.32	25.64	25.52	**25.84**
Overpass	26.14	26.84	26.87	26.89	27.17	27.03	26.89	27.76	27.83	27.87	**28.19**
Parkinglot	23.20	23.46	23.50	23.59	23.72	23.69	24.44	24.11	24.45	24.38	**24.54**
River	29.03	29.13	29.10	29.11	29.14	29.11	28.89	29.28	29.25	29.21	**29.41**
Runway	29.99	30.58	30.68	30.60	30.98	30.79	**32.53**	31.21	31.25	31.19	31.28
Sparseresidential	30.88	31.17	31.28	31.25	31.35	31.29	30.81	31.55	31.57	**31.61**	31.06
Storagetanks	31.67	32.16	32.29	32.29	32.42	32.37	29.62	32.74	32.71	**32.77**	32.63
Tenniscourt	31.28	31.59	31.68	31.74	31.99	31.93	30.76	32.40	32.51	**32.54**	31.64
Average	28.92	29.29	29.42	29.41	29.52	29.47	29.52	29.80	29.92	29.88	**29.97**

**Table 3:** Complexity Analysis of Different Super-Resolution Models on the UC Merced Dataset ×4SR Task.

Method	Scale	Param/M	FLOPs/G	PSNR/dB
SRCNN [12]	×4	0.057	4.53	26.79
LGCNet [30]		0.193	7.12	27.06
IMDN [22]		0.694	13.07	27.23
CTNet [24]		0.413	3.6	27.39
ESRT [23]		0.750	12.77	27.41
DCM [31]		2.18	13.0	27.28
FeNet [32]		0.351	1.44	27.30
ACT [36]		46.1	22	27.54
TransEnet [37]		37.3	21.44	27.81
Ours		2.37	9.60	27.91

**Table 4:** Ablation experiments of each component.

Model	MSPLCK	EPA	MSLA	PSNR/dB	SSIM	FLOPs	Param
Baseline				27.44	0.7488	3.96 G	0.97 M
M0		✓	✓	27.74	0.7626	6.60 G	1.63 M
M1	✓		✓	27.70	0.7611	8.42 G	2.03 M
M2	✓	✓		27.78	0.7633	9.20 G	2.26 M
M3	✓			27.65	0.7585	9.66 G	2.33 M
M4		✓		27.70	0.7618	6.84 G	1.72 M
M5			✓	27.58	0.7578	4.42 G	1.08 M
HSFAN	✓	✓	✓	27.91	0.7696	9.60 G	2.37 M

The symbol “✓” indicates that the corresponding module is used in the model. HSFAN denotes the full Hybrid-Scale Feature Aggregation Network.

**Table 5:** MSPLC module ablation experiments.

Model	GC3 × 3	GC5 × 5	GC7 × 7	PSNR/dB	SSIM	FLOPs	Param
M6	✓			27.72	0.7625	9.48 G	2.34 M
M7		✓		27.76	0.7627	9.58 G	2.37 M
M8			✓	27.82	0.7661	9.73 G	2.40 M
HSFAN	✓	✓	✓	27.91	0.7696	9.60 G	2.37 M

The symbol “✓” indicates that the corresponding module is used in the model. HSFAN denotes the full Hybrid-Scale Feature Aggregation Network.

**Table 6:** EPA module ablation experiments.

Method	CA	PA	SPA	PSNR/dB	SSIM	FLOPs	Param
Series	✓	✓		27.76	0.7640	8.44 G	2.07 M
Series	✓	✓	✓	27.75	0.7632	8.56 G	2.10 M
Parallel	✓	✓	✓	27.91	0.7696	9.60 G	2.37 M

The symbol “✓” indicates that the corresponding module is used in the model.

**Table 7:** MSLA module ablation experiments.

Model	Conv	Attention	Multi-scale	PSNR/dB	SSIM	FLOPs	Param
M9	✓			27.81	0.7662	9.90 G	2.44 M
M10		✓		27.85	0.7662	9.40 G	2.32 M
M11			✓	27.79	0.7647	9.60 G	2.36 M
M12		✓	✓	27.91	0.7696	9.60 G	2.37 M

The symbol “✓” indicates that the corresponding module is used in the model.

## Data Availability

The original contributions presented in this study are included in the article. Further inquiries can be directed to the corresponding author.
